# Response of catecholaminergic neurons in the mouse hindbrain to glucoprivic stimuli is astrocyte dependent

**DOI:** 10.1152/ajpregu.00368.2017

**Published:** 2018-03-28

**Authors:** Richard C. Rogers, David H. McDougal, Sue Ritter, Emily Qualls-Creekmore, Gerlinda E. Hermann

**Affiliations:** ^1^Pennington Biomedical Research Center, Baton Rouge, Louisiana; ^2^Department of Veterinary and Comparative Anatomy, Pharmacology and Physiology, Washington State University, Pullman, Washington

**Keywords:** astrocyte, hindbrain, hypoglycemia

## Abstract

Hindbrain catecholaminergic (CA) neurons are required for critical autonomic, endocrine, and behavioral counterregulatory responses (CRRs) to hypoglycemia. Recent studies suggest that CRR initiation depends on hindbrain astrocyte glucose sensors (McDougal DH, Hermann GE, Rogers RC. *Front Neurosci* 7: 249, 2013; Rogers RC, Ritter S, Hermann GE. *Am J Physiol Regul Integr Comp Physiol* 310: R1102–R1108, 2016). To test the proposition that hindbrain CA responses to glucoprivation are astrocyte dependent, we utilized transgenic mice in which the calcium reporter construct (GCaMP5) was expressed selectively in tyrosine hydroxylase neurons (TH-GCaMP5). We conducted live cell calcium-imaging studies on tissue slices containing the nucleus of the solitary tract (NST) or the ventrolateral medulla, critical CRR initiation sites. Results show that TH-GCaMP5 neurons are robustly activated by a glucoprivic challenge and that this response is dependent on functional astrocytes. Pretreatment of hindbrain slices with fluorocitrate (an astrocytic metabolic suppressor) abolished TH-GCaMP5 neuronal responses to glucoprivation, but not to glutamate. Pharmacologic results suggest that the astrocytic connection with hindbrain CA neurons is purinergic via P2 receptors. Parallel imaging studies on hindbrain slices of NST from wild-type C57BL/6J mice, in which astrocytes and neurons were prelabeled with a calcium reporter dye and an astrocytic vital dye, show that both cell types are activated by glucoprivation but astrocytes responded significantly sooner than neurons. Pretreatment of these hindbrain slices with P2 antagonists abolished neuronal responses to glucoprivation without interruption of astrocyte responses; pretreatment with fluorocitrate eliminated both astrocytic and neuronal responses. These results support earlier work suggesting that the primary detection of glucoprivic signals by the hindbrain is mediated by astrocytes.

## INTRODUCTION

Glucoregulation, the process that maintains plasma glucose concentrations within a critical physiological range, is essential for brain function and survival. Glucose sensors both in the periphery and in the brain monitor glucose availability (6, 51, 60, 73, 75). These glucose sensors detect glucose deficit and activate glucoregulatory responses that restore glucose levels, referred to as counterregulatory responses (CRRs; 13). A critical central nervous system site for the defense against glucoprivation is the caudal medulla (16), which contains at least two glucoregulatory sites: the nucleus of the solitary tract (NST) and the ventrolateral medulla (VLM). The NST is the recipient of vagal afferent projections from peripheral glucose sensors (2, 49, 80) and maintains efferent connections necessary for regulating nutrient homeostasis and digestion (1, 5, 14, 15, 44, 63, 78). The NST and VLM are brain sites important for generation of physiological and behavioral responses to glucose deficit, including increased feeding and elevation of circulating glucagon, corticosteroids, and epinephrine (3, 39, 62). In addition, reduced glucose availability triggers dramatic acceleration of gastric motility, a response that increases availability of ingested carbohydrate for glucose absorption (9, 13, 29, 70).

Hindbrain catecholaminergic (CA) neurons are critically involved in glucoregulatory functions. Selective immunotoxin lesions, gene silencing, chemogenetic activation, and localized glucoprivation have revealed that activation of these neurons is necessary for elicitation of feeding, corticosterone secretion, and elevation of adrenal medullary secretion in response to glucose deficit (7, 33, 34, 59, 62). Additionally, CA NST neurons may be involved in producing the increased gastric motility evoked by glucose deficit (29, 66, 67).

Whether any hindbrain CA neurons are themselves glucose sensing remains under investigation. However, several lines of evidence implicate astrocytes in brain glucose sensing and glucoregulation. Mice lacking the gene for the type 2 glucose transporter (GLUT2), a glucose transporter of particular importance for astrocytes (71), do not increase glucagon secretion in response to hypoglycemia as wild-type mice do. However, selective reexpression of GLUT2 in astrocytes alone “rescues” this CRR (10, 38).

Imaging studies have demonstrated that NST astrocytes increase cytoplasmic calcium (Ca^++^) in response to decreased glucose concentration and to 2-deoxyglucose (2DG), which reduces cellular glucose utilization (40). Recent in vivo results have also shown that astrocyte inactivation blocks the increase in gastric motility stimulated by glucose deficit (29). Furthermore, physiological studies have suggested that medullary astrocytes are also necessary for the hyperglycemic response to hindbrain cytoglucopenia. Fourth ventricular administration of 2DG to anesthetized rats increased plasma glucose levels. Preexposure of the fourth ventricle to fluorocitrate, a blocker of astrocytic metabolism, suppressed the glucoprivation-induced increase in blood glucose levels (66).

Taken together, these data suggest that astrocytes are glucose sensors and that detection of a low-glucose state by brain stem astrocytes could, in turn, activate neurons, including CA neurons that control CRR initiation. Because of their critical importance for glucoregulation, CA neurons were targeted for the present live cell calcium-imaging study. This study was designed to determine *1*) whether preidentified/labeled CA neurons in the NST or VLM are activated by a glucoprivation challenge, *2*) whether the CA neuron response to this stimulus is dependent on functional astrocytes, and *3*) whether potential gliotransmitter(s) (e.g., purinergic or glutamatergic) might be identified (21, 55).

## MATERIALS AND METHODS

### Animals

The first series of live cell calcium-imaging studies used adult C57BL/6J mice (males and females; body weight 18–28 g). The second series of imaging experiments used adult male and female transgenic C57BL/6J mice that had been bred to selectively express a genetically encoded calcium indicator [GECI; initially developed by J. Nakai et al. (47)] to be associated with CA neurons. The advantage of GECIs is that they can be genetically specified for studies in living organisms. GCaMP5 is a GECI, created from a fusion of green fluorescent protein (GFP), calmodulin, and M13, a peptide sequence from myosin light chain kinase. In our studies, transgenic mice expressing a Cre recombinase-inducible variant of the fluorescent calcium indicator protein GCaMP5 (JAX cat. no. 24477; Jackson Laboratory) were crossed to TH-Cre transgenic mice [which have the rat tyrosine hydroxylase (TH) promoter driving expression of Cre recombinase in CA cells; JAX cat. no. 08601] and are referred to in these studies as “TH-GCaMP5.” All animals used in these studies were obtained from our breeding colonies at Pennington Biomedical Research Center. Genotyping data and breeding records were collected and managed using Research Electronic Data Capture (REDCap) tools hosted at Pennington Biomedical Research Center (25). Animals were maintained in a room with a 12-h light-dark cycle with constant temperature and humidity and had access to food and water ad libitum. All experimental protocols were approved by the Institutional Animal Care and Use Committees of Pennington Biomedical Research Center and were performed according to the guidelines determined by the National Institutes of Health.

### Live Cell Calcium Imaging: Prelabeling of Cells and Preparation of Hindbrain Slices

Live cell calcium-imaging studies were done on ex vivo slices of the mouse hindbrain to determine whether NST neurons and/or astrocytes of that species could detect hypoglycemic conditions. The first series of studies were done in hindbrain slices from C57BL/6J mice. Neurons and astrocytes were prelabeled via incubation with calcium reporter dye Cal-520 (AAT Bioquest), and astrocytes were identified by specific uptake of biomarker sulfarhodamine 101 (SR101, astrocyte vital dye; Sigma-Aldrich, St. Louis, MO; 41). Medullary slices were prepared from adult male and female C57BL/6J mice (*n* = 12; body weight 20–28 g). Animals were anesthetized with urethane. After decapitation and swift removal of hindbrain to cold (~4°C) carbogenated (95% O_2_, 5% CO_2_) cutting solution (see recipe below), 300-µm-thick slices were cut coronally through the medulla with a sapphire knife on a vibratome and placed in a scintillation vial containing carbogenated normal Krebs solution (recipe below). Slices were prelabeled by incubation in a small plastic petri dish containing 3 ml normal carbogenated Krebs solution plus 50 µg Cal-520 (AAT Bioquest) and 0.25 µg SR101 (Sigma-Aldrich) dissolved in 50 µl pluronic-DMSO. Incubation with dyes took place at 29°C for ~30 min (27).

Labeled brain slices were returned to a scintillation vial containing carbogenated normal Krebs solution before imaging. Hindbrain slices containing labeled neurons (Cal-520 uptake only) and astrocytes (Cal-520 + SR101 uptake) were placed in the recording chamber of a Zeiss Axioscope fixed-stage upright microscope. Slices were continuously perfused with the carbogenated, normal Krebs recording solution (33°C; 2.5 ml/min flow rate).

The second series of live cell calcium-imaging experiments used male and female TH-GCaMP5 transgenic mice (*n* = 48). These transgenic mice selectively express GCaMP5 in CA neurons of the NST and VLM; ex vivo hindbrain slices contained specifically prelabeled CA neurons. Harvesting and slicing the hindbrain sections proceeded as described above. No further prelabeling of slices was performed; hindbrain sections were directly transferred to a scintillation vial containing carbogenated normal Krebs solution before imaging. Similar to the description of imaging above, hindbrain sections containing prelabeled TH-GCaMP5 NST (or VLM) neurons were placed in the recording chamber and were continuously perfused with the carbogenated, normal Krebs recording solution.

### Perfusion Solutions

All solutions were freshly prepared on the day of the experiment. Cutting solution contained 110 mM choline chloride, 25 mM NaHCO_3_, 2.5 mM KCl, 7 mM MgSO_4_·7H_2_O, 1.25 mM NaH_2_PO_4_, 10 mM glucose, and 0.5 mM CaCl_2_·2H_2_O. Normal Krebs solution contained 124 mM NaCl, 25 mM NaHCO_3_, 3.0 mM KCl, 1 mM MgSO_4_·7H_2_O, 1.5 mM NaH_2_PO_4_, 5 mM glucose, and 1.5 mM CaCl_2_·2H_2_O.

Each candidate antagonist (see Table 1) was dissolved in the normal Krebs solution and contained in its separate perfusion flask. These pretreatments were applied to the slice for 20 min via perfusion through the recording chamber. Additionally, 1 mM glutamate dissolved in normal Krebs solution was used as a cell viability test.

**Table 1.** Pretreatment groups

Name	Abbreviation	Action	Source	Concentration Used, µM	Reference No.
Fluorocitrate	FC	Selective inhibitor of glial metabolism	Sigma-Aldrich	200	74
Suramin		Broad spectrum purinergic P2 receptor antagonist	Tocris	100	74
*dl*-2-Amino-5-phosphonopentanoic acid	DL-AP5	Potent NMDA receptor antagonist	Tocris	200	74
Pyridoxalphosphate-6-azophenyl-2′,5′-disulfonic acid	PPAD	P2X purinoceptor antagonist	Tocris	10	11
5-{[5-(2,8-Dimethyl-5H-dibenzo[*a*,*d*]cyclohepten-5-yl)-3,4-dihydro-2-oxo-4-thioxo-1(2H)-pyrimidinyl]methyl}-*N*-2H-tetrazol-5-yl-2-furancarboxamide	AR-C (AR-C 118925XX)*	Selective, competitive P2Y_2_ receptor antagonist	Tocris	10	57
4,4′-{Carbonylbis[imino-3,1-(4-methyl-phenylene)carbonylimino]}bis(naphthalene-2,6-disulfonic acid) tetrasodium salt	NF 340	P2Y_11_ antagonist	Tocris	10	17
2-(2-Furanyl)-7-[3-(4-methoxyphenyl)propyl]-7H-pyrazolo[4,3-e][1,2,4]triazolo[1,5-c]pyrimidin-5-amine	SCH (SCH442416)*	Selective adenosine A_2A_ receptor antagonist	Tocris	10	69

NMDA, *N*-methyl-d-aspartate; P2Y_2_ and P2Y_11_, P2Y purinoceptors 2 and 11, respectively.

* Solubilized in DMSO, then diluted in Krebs.

Composition of the “low-glucose/glucoprivic challenge” solution (LG/2DG) was identical to the normal Krebs solution with the exception that glucose concentration was only 1 mM with the addition of 4 mM 2-deoxy-d-glucose (2DG; Sigma-Aldrich).

### Perfusion Chamber and Solution Delivery

Individual brain slices were transferred to the temperature-regulated perfusion chamber [refer to Rogers et al. (65) for specific details regarding construction of chamber and thermocontrol]. The recording chamber was continuously perfused at a rate of 2.5 ml/min with carbogenated Krebs (or experimental solutions) warmed to 33°C. Solutions were carbogenated in individual perfusion flasks; solenoid valves (ValveLink 8; Automate Scientific, San Francisco, CA) were used to select perfusates to be directed to the slice. Perfusates were delivered through a manifold (Small Parts, Miami Lakes, FL) to a roller pump (Dynamax; Rainin, Woburn, MA) to switch between the normal bathing solution and the challenge or experimental solutions without interruption to perfusion of the brain slice being studied. Activation of individual solenoid valves to switch perfusion solutions served as *time 0* for recording purposes. The length of the common perfusion line coming into the chamber required a total of 45 s to make the switch of perfusion solutions complete.

### Live Cell Calcium Imaging and Experimental Design

Prelabeled slices (either Cal-520 + SR101 dyes in C57BL/6J or TH-GCaMP5 transgenics) were transferred to a custom imaging chamber (64, 65); as described above. Hindbrain slices were viewed with a Zeiss Axioscope 2 fixed-stage microscope equipped with normal epifluorescence optics as well as with a Yokogawa CSU21 laser confocal scan head and Hamamatsu ORCA-ER camera. Prelabeled cells of interest were selected visually with the epifluorescence optics, and then confocal images were captured with the ORCA-ER.

It was our intention to record glucoprivic responses in neurons and astrocytes in both the NST and VLM. Whereas the observation of glucoprivic responses in the NST in adult mice was straightforward, such recordings in the VLM were made difficult because of the heavy concentration of myelinated pathways in this region of the medulla. The reflective nature of myelin obscures all but a few labeled cells in the VLM. This mirrors our earlier experiences of calcium imaging of reticular neurons of the medulla (48). As a result, only limited observations of TH-GCaMP5 VLM neurons were possible.

#### Imaging of NST neurons and astrocytes.

**Fig. 1.**
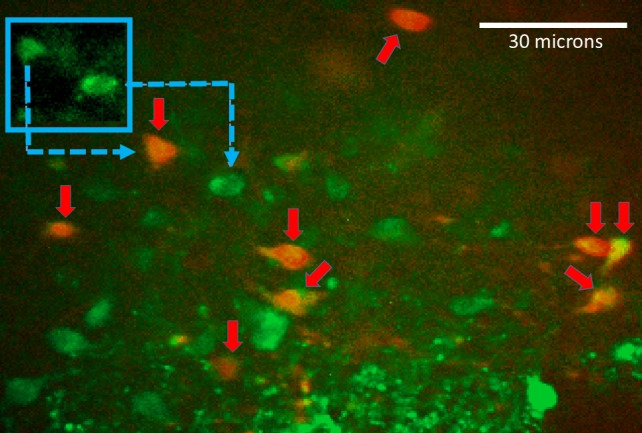
Image of a confocal field of view with nucleus of the solitary tract astrocytes and neurons labeled by incubation with the calcium reporter dye Cal-520 (green) and the astrocyte vital stain sulfarhodamine 101 (SR101, red). *Inset* shows that both astrocytes and neurons are labeled with Cal-520 (green) but only astrocytes are revealed with SR101. Prelabeling with Cal-520 and SR101 allows for discrimination of astrocytes and neurons at the cellular level by comparing images captured using the 488- and 561-nm laser lines. At 488 nm, both astrocytes and neurons (i.e., prelabeled with Cal-520) will appear green; at 561 nm, only astrocytes will also appear red (40, 41). A single, dual-exposure image was collected just before and following each experimental trial to confirm the cell types being recorded.

Prelabeling with Cal-520 and SR101 allows for discrimination of astrocytes and neurons at the cellular level by comparing images captured using the 488-nm and 561-nm excitation. At 488 nm, both astrocytes and neurons (i.e., prelabeled with Cal-520) will appear green (509 nm emission); at 561 nm, only astrocytes will also appear red (605 nm emission) (40, 41). A single, dual-exposure image was collected just before and after each experimental trial to confirm the cell types being recorded (Fig. 1). Once in the recording chamber, hindbrain slices were perfused with normal Krebs solution for a minimum of 10 min. Slices were then challenged with the low-glucose/glucoprivic challenge of Krebs with LG/2DG (300 s). [Although we have previously recorded increased astrocyte calcium signaling in response to either glucoprivic stimulus alone (40), pilot studies revealed that the changes occurred more quickly with the combined stimulation (i.e., activation within 3–5 min for low glucose + 2DG vs. 10–20 min with either stimulation alone).] Changes in intracellular calcium concentrations within Cal-520-prelabeled NST astrocytes and NST neurons in response to different stimuli were recorded using the 488-nm laser line to excite the Cal-520. Increases in intracellular calcium concentrations are reflected as an increase in fluorescence and are indicative of increased cellular activity.

During experimental trials, time-lapse images of mixed fields of NST astrocytes and neurons were monitored for their responses to the glucoprivic conditions. Additional prelabeled hindbrain slices were first exposed to either fluorocitrate [FC; to temporarily block astrocytic metabolic activity (66, 74)] or suramin (to block purinergic receptor activity) before glucoprivic challenges. After an additional 10-min washout with normal Krebs solution, slices were challenged with 1 mM glutamate to verify that any absence or reduction in response activity to the glucoprivic challenge was specific to the blocking agent and not due to cell damage. Thus, demonstration of cellular viability (74) required an increase in fluorescence in response to glutamate challenge of at least 10% above baseline levels. Time-lapse laser confocal images of changes in intracellular calcium levels of both astrocytes and neurons in response to challenges were captured with the ORCA-ER at a rate of one frame per second.

#### Imaging of TH-GCaMP5 neurons in the NST.

In the second group of imaging studies, changes in intracellular calcium concentrations in TH-GCaMP5 NST neurons were monitored via the fluorescent calcium indicator protein (GCaMP5) using the same 488-nm excitation/509-nm arrangement, as above. During experimental trials, time-lapse images of these TH-GCaMP5 NST neurons were monitored for their responses to the glucoprivic conditions with and without pretreatment with different pharmacological agents to determine the pathway of communication between NST astrocytes and CA neurons involved in sensing low-glucose conditions.

Once in the recording chamber, hindbrain slices were perfused with normal Krebs solution for a minimum of 10 min. Slices were then challenged with the glucoprivic challenge of Krebs with LG/2DG (300 s). Normal Krebs solution was resumed to wash out the glucoprivic stimulus. As described above, glutamate (1 mM) was bath applied (60 s) to verify the viability of the prelabeled NST cells as reflected by their ability to respond with calcium signals. Only those neurons that passed the viability test of responding to glutamate stimulation were included in our analyses regarding neuronal responsiveness to LG/2DG.

#### Selection of putative gliotransmitter antagonists.

CA neurons are activated by glucoprivic stimulation (61, 62). This activation is associated with the expression of c-fos, which, in turn, is associated with sustained increases in intracellular calcium (45) Therefore, we targeted gliotransmission pathways likely to produce increases in intracellular calcium in target neurons.

Previous studies have identified potential candidates for gliotransmitters to include glutamate and purines (4, 18, 28, 35, 37, 52, 53, 74). Therefore, to determine the pathway of communication between hindbrain astrocytes and CA neurons involved in sensing low-glucose conditions, some slices were pretreated with one of the solutions listed in Table 1 for 20 min via perfusion through the recording chamber. Pretreated hindbrain slices were then challenged with low-glucose conditions, and viability of prelabeled TH-GCaMP5 neurons cells was then verified by exposing slices to glutamate via the perfusion bath, as described above.

Suramin was chosen because of the potential for astrocytic purine release onto neuronal P2 receptors to activate neurons through G_q_ protein-mediated release of endoplasmic reticular (ER) calcium stores (76). AR-C (5-{[5-(2,8-dimethyl-5H-dibenzo[*a*,*d*]cyclohepten-5-yl)-3,4-dihydro-2-oxo-4-thioxo-1(2H)-pyrimidinyl]methyl}-*N*-2H-tetrazol-5-yl-2-furancarboxamide) selectively targets P2Y purinoceptor 2 (P2Y_2_) ATP receptors coupled with G_q_ protein-mediated release of ER calcium stores (57). NF 340 (4,4′-{carbonylbis[imino-3,1-(4-methyl-phenylene)carbonylimino]}bis(naphthalene-2,6-disulfonic acid) tetrasodium salt) selectively inhibits P2Y_11_, another ATP receptor coupled to G_q_ protein-mediated release of ER calcium stores (17, 43).

SCH442416 {2-(2-furanyl)-7-[3-(4-methoxyphenyl)propyl]-7H-pyrazolo[4,3-e][1,2,4]triazolo[1,5-c]pyrimidin-5-amine}, an A_2A_ receptor antagonist, blocks one of the few excitatory mechanisms of calcium store release driven by adenosine [another potential gliotransitter (69)]. DL-AP5 (*dl*-2-amino-5-phosphonopentanoic acid) antagonism of *N*-methyl-d-aspartate (NMDA) receptors has been shown to block glutamatergic communications between astrocytes and NST neurons. NMDA receptors are ligand-gated cation channels that, when activated, produce robust increases in intracellular calcium (17, 43, 57, 69, 74, 76).

Finally, our previous studies suggested the involvement of ATP as a gliotransmitter between low glucose-sensing astrocytes and hindbrain neurons (66). Therefore, we added one more experimental group to directly observe the effects of ATP on CA neurons and whether these neurons retained their ability to respond to ATP even in the presence of FC pretreatment.

#### Imaging of TH-GCaMP5 neurons in the VLM.

Because of the physical difficulties in imaging identified cells in this region, TH-GCaMP5 VLM neurons were only evaluated for their responses to the LG/2DG glucoprivic stimulus under control, fluorocitrate, or suramin pretreatment of hindbrain slices. Studies were conducted using the same protocol as described for the NST preparations. Again, only neurons that passed the viability test of responding to glutamate stimulation were included in our analyses regarding neuronal responsiveness to LG/2DG.

### Data and Statistical Analysis of Cellular Responses in NST

Nikon Elements AR software was used to analyze the confocal live cell fluorescent signals in astrocytes and/or neurons as previously described (28). Individual astrocytes and neurons were designated as “regions of interest,” and their fluorescence signal over time was captured. Background fluorescence was subtracted from the cellular fluorescence signal. The relative changes in cytoplasmic calcium in the cells were expressed as changes in fluorescence [(Δ*F/F*)%], where *F* is the intensity of the baseline fluorescence signal before stimulation and Δ*F* is the difference between the peak fluorescence intensity and the baseline signal of each individual region of interest.

Data from Cal-520 + SR101-prelabeled astrocytes (*n* = 151) in the C57BL/6J mice in response to either LG/2DG or glutamate challenge under control, FC, or suramin pretreatment conditions were evaluated for statistical significance via one-way ANOVA and Dunnett’s multiple-comparison post hoc tests. Significance was set at *P* < 0.05. All data are reported as means ± SE.

Similarly, data from Cal-520-labeled neurons (*n* = 176) in the C57BL/6J mice in response to either LG/2DG or glutamate challenge under control, FC, or suramin pretreatment conditions were evaluated for statistical significance via one-way ANOVA and Dunnett’s multiple-comparison post hoc tests. Significance was set at *P* < 0.05. All data are reported as means ± SE.

Data related to start times of response to LG/2DG stimulation from Cal-520 + SR101-prelabeled astrocytes and neurons in the C57BL/6J mice were evaluated for statistical significance using unpaired *t*-tests. Data are reported as means ± SE; significance was set at *P* < 0.05.

The proportion of viable, general population NST neurons also responsive to LG/2DG was compared with the proportion of viable, specifically identified TH (i.e., TH-GCaMP5) NST neurons also responsive to glucoprivic stimulation using χ^2^-analysis. Significance was set at *P* < 0.05.

Data from TH-GCaMP5 NST neurons (*n* = 142) responding to LG/2DG challenge after various pretreatment conditions were evaluated for statistical significance using one-way ANOVA and Dunnett’s multiple-comparison post hoc tests. Again, significance was set at *P* < 0.05; all data are reported as means ± SE.

Finally, data regarding responsiveness of TH-GCaMP5 NST neurons (*n* = 24) to ATP, whether or not they had been preexposed to fluorocitrate, were evaluated for statistical significance using *t*-tests. Data are reported as means ± SE; significance was set at *P* < 0.05.

### Data and Statistical Analysis of Neuronal Responses in VLM

Because of the scarcity of available identified cells, TH-GCaMP5 VLM neurons (*n* = 34) were only evaluated for their responses to the LG/2DG glucoprivic stimulus under control, fluorocitrate, or suramin pretreatment of hindbrain slices. Data from TH-GCaMP5 VLM neurons responding to LG/2DG challenge after various pretreatment conditions were evaluated for statistical significance using one-way ANOVA and Dunnett’s multiple-comparison post hoc tests. Significance was set at *P* < 0.05; all data are reported as means ± SE.

### Immunohistochemical Verification of Association of GCaMP5 With TH Neurons

TH-GCaMP5 transgenic mice (*n* = 3) were deeply anesthetized via inhalation of isoflurane and transcardially perfused with ice-cold saline followed by 10% formalin. Brains were harvested, postfixed in 10% formalin for 24 h, and then transferred into 30% sucrose for cryopreservation until sectioning (30 µm per section) throughout the brain using a sliding microtome. Brain sections were processed using free-floating immunohistochemistry to analyze the coexpression of TH in neurons expressing the calcium indicator GCaMP. Sections were blocked in 3% normal donkey serum, followed by incubation in primary antibodies, rabbit anti-tyrosine hydroxylase (1:1,000, AB-152; Millipore) and chicken anti-GFP (1:1,000, ab-13970; Abcam; GFP is a component of GCaMP5), and detected with fluorescent-labeled secondary antibodies Alexa Fluor 594 donkey anti-rabbit (1:200, A-21207; Thermo Fisher Scientific) and Alexa Fluor 488 donkey anti-chicken (1:200, 703-546-155; Jackson ImmunoResearch Laboratories), respectively. Histological sections from each mouse at the level of the area postrema were examined microscopically. Cells within the NST demonstrating antibody label for TH, GFP, or both labels were counted and photographed.

## RESULTS

### Responses to LG/2DG of NST Astrocytes and Neurons From C57BL/6J Mice (Prelabeled via SR101 and Cal-520)

**Fig. 2.**
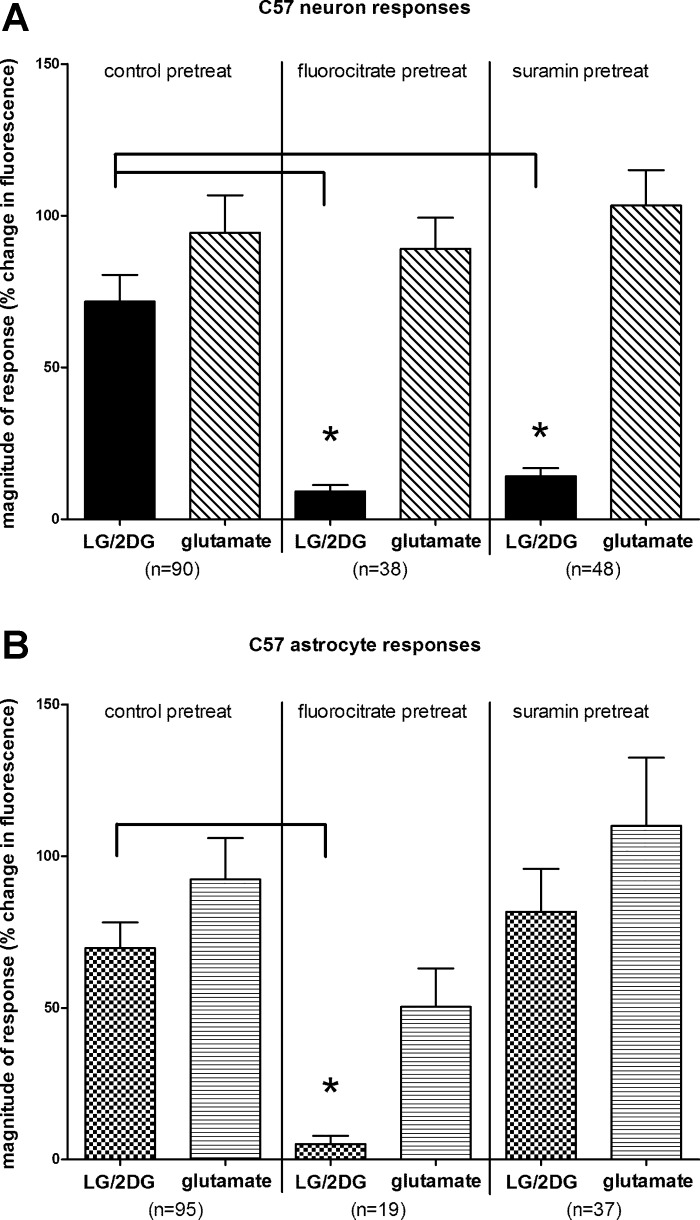
*A*: magnitude of peak changes in fluorescence due to intracellular calcium fluxes in nucleus of the solitary tract (NST) neurons from C57BL/6 (C57) mouse brain slice in response to glucoprivic stimuli [low glucose/2-deoxyglucose (LG/2DG)] or glutamate (1 mM). Under control conditions, glucoprivation activated NST neurons to approximately the same extent as glutamate, which provides a robust test of cell viability. Fluorocitrate, a selective inhibitor of astrocyte signaling, eliminated the NST neuron response to glucoprivic stimulation while having no effect on NST neuron responses to glutamate. Thus, fluorocitrate does not cause a general depression of NST neuron activity. Similarly, suramin, a P2 purinergic antagonist, blocked the effects of glucoprivic stimulation while having no effect on the NST neuron response to glutamate. ANOVA regarding neuronal responsiveness to LG/2DG: *F*_2,170_ = 21.99, *P* < 0.0001; *Dunnett’s multiple-comparison post hoc test: *P* < 0.05. ANOVA regarding neuronal responsiveness to glutamate: *F*_2,141_ = 0.3389, *P* = 0.7131. *B*: astrocyte calcium signals generated in Cal-520- and SR-101-colabeled astrocytes in the NST slices from the C57BL/6 mouse. As in the case with NST neurons, astrocytes produced a robust response to LG/2DG stimulation on par with that produced by 1 mM glutamate under control conditions. As expected, fluorocitrate eliminated the LG/2DG response (ANOVA: *F_2_*_,144_ = 6.71, *P* = 0.0016; *Dunnett’s multiple-comparison post hoc test: *P* < 0.05). Astrocyte responsiveness to glutamate is reduced under fluorocitrate treatment, but the reduction is not significant (ANOVA: *F*_2,95_ = 2.02, *P* = 0.1380). Unlike the case for NST neurons, suramin had no effect on astrocyte responsiveness to LG/2DG. These results suggest that LG/2DG activation of NST neurons is dependent on primary NST astrocyte detection and purinergic signaling. Pretreat, pretreatment.

These live cell imaging studies allowed us to simultaneously monitor the responses of both astrocytes and neurons in the NST to the LG/2DG challenges. Under normal, control conditions, 60 of the 90 (~67%) viable, prelabeled NST neurons responded to LG/2DG challenge. [An individual cell was considered to have responded to a given stimulus if it induced a peak change in (Δ*F*/*F*)% >10% above baseline (28, 41, 50, 68).] Under these same conditions, 70 of the 95 (~74%) viable, prelabeled NST astrocytes responded to LG/2DG challenge. Note that the magnitudes of response by either NST astrocytes or NST neurons to this glucoprivic challenge are fairly robust and similar in magnitude of response to stimulation by 1 mM glutamate in the perfusion bath (Fig. 2).

**Fig. 3.**
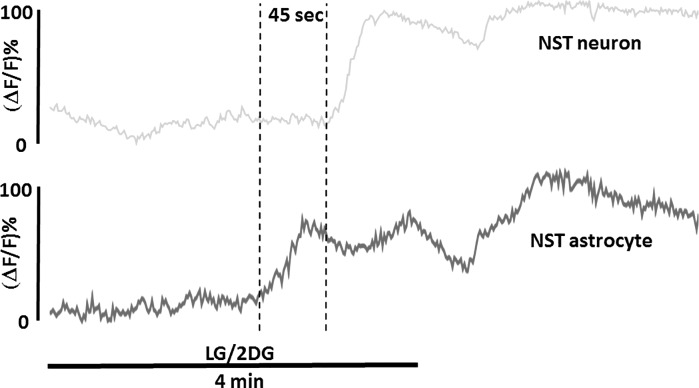
Plots of the calcium-induced fluorescence signals evoked in an astrocyte (dark gray curve) and closely adjacent nucleus of the solitary tract (NST) neuron (light gray curve) evoked by exposure to a low-glucose/2-deoxyglucose (LG/2DG) challenge. On average, NST astrocytes responded ~40 s before neurons. Similar sequential patterns of activation have also been observed for hindbrain astrocyte-neuron interactions that regulate responses to thrombin (74) and hypoxia (58). Δ*F/F*, change in fluorescence intensity relative to baseline.

Given that both prelabeled cell types were present in the same microscopic field of the hindbrain slice, it was possible to record relative time of onset to response to the LG/2DG challenge. On average, NST neurons responded within 206.6 ± 10.2 s after the bath perfusion was changed whereas NST astrocytes responded within 164.2 ± 8.1 s of the challenge. This difference in start time of nearly 40 s was statistically significant [*t* = 3.298, degrees of freedom = 128, *P* = 0.0013]. Thus, these studies demonstrated that both NST astrocytes and NST neurons are activated by glucoprivic conditions but, on average, astrocytes responded first by ~40 s (Fig. 3).

Pretreatment of hindbrain slices with the astrocytic metabolic blocker, FC, resulted in blocking both astrocytic and, presumably subsequent, neuronal responses to LG/2DG challenge (Fig. 2). FC, a selective inhibitor of astrocyte signaling, eliminated the NST neuron response to glucoprivic stimulation while having no effect on NST neuron responses to glutamate. Thus, FC does not cause a general depression of NST neuron activity. Similarly, suramin, a P2 purinergic antagonist, blocked the effects of glucoprivic stimulation while having no effect on the NST neuron response to glutamate. [ANOVA regarding neuronal responsiveness to LG/2DG: *F*_2,170_ = 21.99, *P* < 0.0001; Dunnett’s multiple-comparison post hoc test: *q* (FC) = 5.42, *q* (suramin) = 5.39, *P* < 0.05; ANOVA regarding neuronal responsiveness to glutamate: *F_2_*_,141_ = 0.3389, *P* = 0.7131.]

As in the case with NST neurons, astrocytes produced a robust response to LG/2DG stimulation on par with that produced by 1 mM glutamate under control conditions. As expected, FC eliminated the astrocytic responsiveness to LG/2DG response (ANOVA: *F*_2,144_ = 6.71, *P* = 0.0016; Dunnett’s multiple-comparison post hoc test: *q* (FC) = 3.31, *P* < 0.05). Astrocyte responsiveness to glutamate is reduced under FC treatment, but the reduction is not significant (ANOVA: *F*_2,95_ = 2.02, *P* = 0.1380). Unlike the case for NST neurons, suramin had no effect on astrocyte responsiveness to LG/2DG.

### Responses of Phenotypically Identified NST Neurons to LG/2DG in TH-GCaMP5 Transgenic Mice

**Fig. 4.**
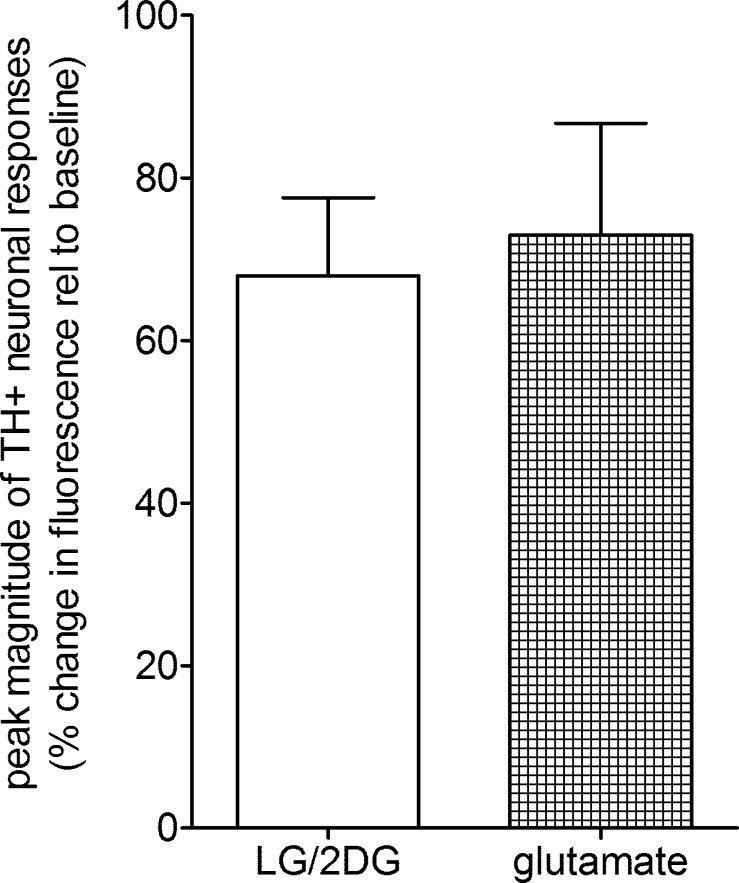
Recordings from tyrosine hydroxylase (TH)-GCaMP5-expressing neurons (*n* = 29) in the nucleus of the solitary tract show a robust calcium-induced fluorescence response to low-glucose/2-deoxyglucose (LG/2DG) glucoprivic stimulus that is not different from a maximal activation elicited by 1 mM glutamate (*P* = 0.76) (% change in fluorescence relative to baseline).

**Fig. 5.**
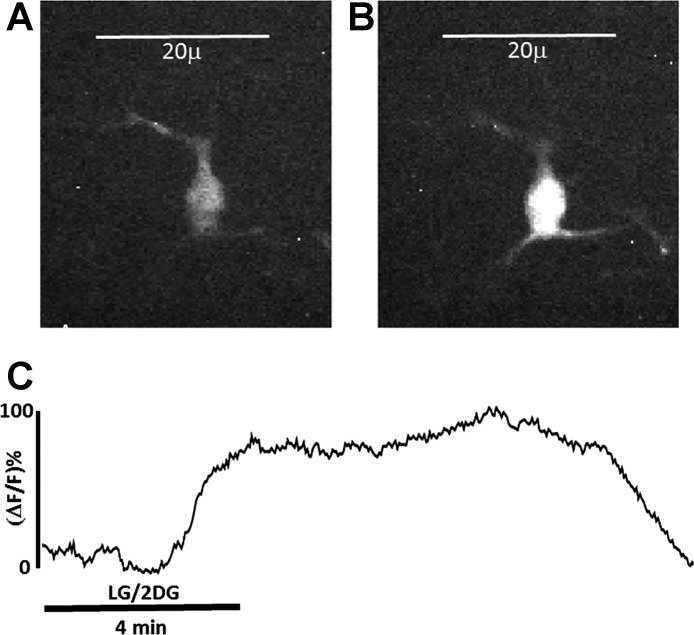
Live cell calcium image of tyrosine hydroxylase (TH)-GCaMP5 neuron in the nucleus of the solitary tract responding to a 4-min LG/2DG glucoprivic challenge. *A*: TH^+^ neuron at rest; beginning of the recording. *B*: same neuron following LG/2DG challenge. *C*: real-time plot of the calcium response of the TH-GCaMP5 neuron pictured in *A* (at rest) and *B* (at peak of activation). Δ*F/F*, change in fluorescence intensity relative to baseline.

**Fig. 6.**
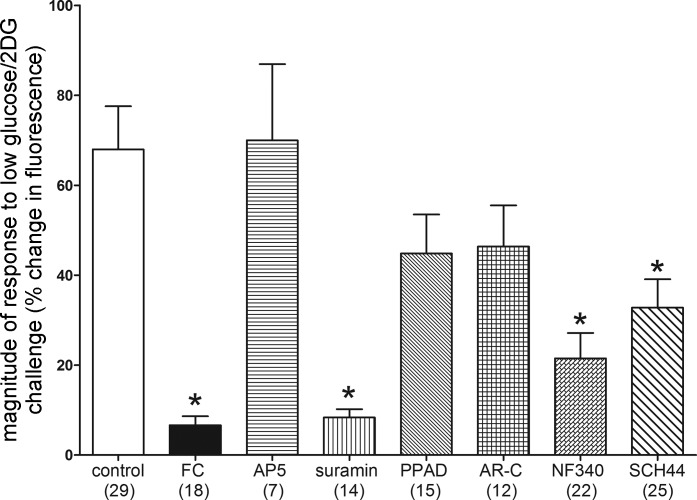
Magnitude of changes in fluorescence due to intracellular calcium fluxes in tyrosine hydroxylase (TH)-GCaMP5 nucleus of the solitary tract (NST) neurons in response to glucoprivic challenge after specific pretreatment conditions (number of neurons studied per each group is noted in parentheses). Exposure of TH-GCaMP5 neurons in hindbrain slices to the various pretreatment conditions produced significant differences in response to subsequent glucoprivic challenge (ANOVA *F*_7,134_ = 8.830, *P* < 0.0001). Similar to the responses seen in the general population of NST neurons (Fig. 2*A*), TH-GCaMP5 NST neurons were robustly activated by glucoprivation, and that effect is essentially blocked by pretreatment with fluorocitrate (FC; Dunnett’s post hoc test: *q* = 6.022, *P < 0.05). The *N*-methyl-d-aspartate antagonist, DL-AP5 (AP5), had no effect to inhibit the TH-GCaMP5 NST neuron response to glucoprivation. However, the nonselective P2 antagonist, suramin, also blocked the responses to low-glucose/2-deoxyglucose (2DG; Dunnett’s post hoc test: *q* = 5.398, *P < 0.05). Finally, both NF 340 (P2Y purinoceptor 11 antagonist) and SCH442416 (SCH44; A_2A_ antagonist) suppressed TH-GCaMP5 NST neuronal responses to glucoprivic conditions (Dunnett’s post hoc test: *q* = 4.843 and 3.799, respectively, *P < 0.05). AR-C, 5-{[5-(2,8-dimethyl-5H-dibenzo[*a*,*d*]cyclohepten-5-yl)-3,4-dihydro-2-oxo-4-thioxo-1(2H)-pyrimidinyl]methyl}-*N*-2H-tetrazol-5-yl-2-furancarboxamide; DL-AP5, *dl*-2-amino-5-phosphonopentanoic acid; NF 340, 4,4′-{carbonylbis[imino-3,1-(4-methyl-phenylene)carbonylimino]}bis(naphthalene-2,6-disulfonic acid) tetrasodium salt; PPAD, pyridoxalphosphate-6-azophenyl-2′,5′-disulfonic acid; SCH442416, 2-(2-furanyl)-7-[3-(4-methoxyphenyl)propyl]-7H-pyrazolo[4,3-e][1,2,4]triazolo[1,5-c]pyrimidin-5-amine.

TH-GCaMP5 NST neurons demonstrated a robust response to the LG/2DG challenge similar to that elicited by glutamate stimulation (Fig. 4). Approximately 90% (26/29) of the viable TH-GCaMP5 NST neurons also increased intracellular calcium in response to the glucoprivic challenge (Figs. 4, 5, and 6). This is in contrast to ~69% (60/90) of the general population of NST neurons observed in the stock C57BL/6J mice. A χ^2^-analysis revealed that this is a significant difference in the proportion of responsive neurons (χ^2^ = 8.08, *P* = 0.0045). This difference is probably a reflection of the fact that the CA neurons are a select and sensitive subpopulation of cells mixed together with many other phenotypes in the NST that are not responsive to glucoprivic stimuli.

Exposure of TH-GCaMP5 NST neurons to the various pretreatment conditions produced significant differences in response to subsequent glucoprivic challenge (ANOVA: *F*_7,134_ = 8.830, *P* < 0.0001). Similar to the responses seen in the general population of NST neurons (Fig. 2*A*), TH-GCaMP5 NST neurons were robustly activated by glucoprivation, and that effect is essentially blocked by pretreatment with FC (Fig. 6; Dunnett’s multiple-comparison post hoc test: *q* = 6.022, *P* < 0.05). The NMDA antagonist (DL-AP5) had no effect to inhibit the TH-GCaMP5 NST neuron response to glucoprivation. However, the nonselective P2 antagonist (suramin) also blocked the responses to LG/2DG (Dunnett’s multiple-comparison post hoc test: *q* = 5.398, *P* < 0.05). Finally, both NF 340 (P2Y_11_ antagonist) and SCH442416 (A_2A_ antagonist) suppressed TH-GCaMP5 NST neuronal responses to glucoprivic conditions (Dunnett’s multiple-comparison post hoc test: *q* = 4.843 and 3.799, respectively, *P* < 0.05).

**Fig. 7.**
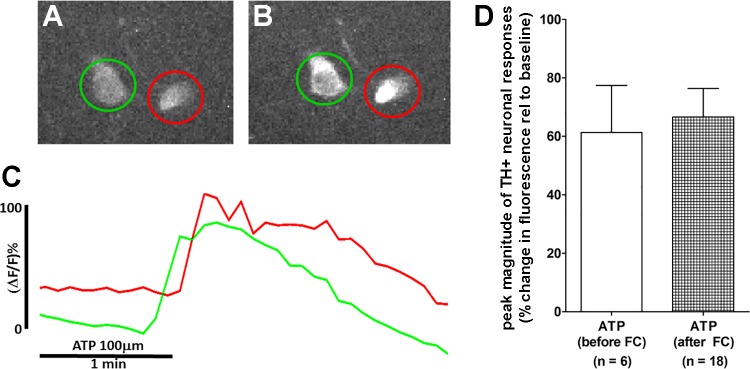
ATP activates tyrosine hydroxylase (TH)-GCaMP neurons in the nucleus of the solitary tract. *A* and *B*: screen shot images of two TH-GCaMP neurons before ATP challenge (*A*) and at the peak of the effect of ATP (*B*). *C*: plot of the kinetics of the calcium-induced fluorescence signal evoked by ATP in the TH-GCaMP neurons. *D*: magnitude of peak changes in fluorescence due to intracellular calcium fluxes in TH-GCaMP neurons responding to ATP. Regardless of whether the medullary slices were pretreated with fluorocitrate (FC), TH-GCaMP neurons still responded with an ~60% increase in calcium signal over baseline. The response profile of the TH-GCaMP neuron circled in red in *A* and *B* is displayed as the red trace in *C*. The same applies to the neuron circled in green in *A* and *B*; its response profile in *C* is the green trace. Δ*F/F*, change in fluorescence intensity relative to baseline.

Finally, exposure of TH-GCaMP5 NST neurons to ATP produced a significant elevation in cytoplasmic calcium, regardless of whether the ATP was delivered with or without FC pretreatment (Fig. 7). In other words, TH-GCaMP5 NST neurons retained their ability to respond to ATP even in the presence of FC pretreatment.

### Responses of Phenotypically Identified VLM Neurons to LG/2DG in TH-GCaMP5 Transgenic Mice

**Fig. 8.**
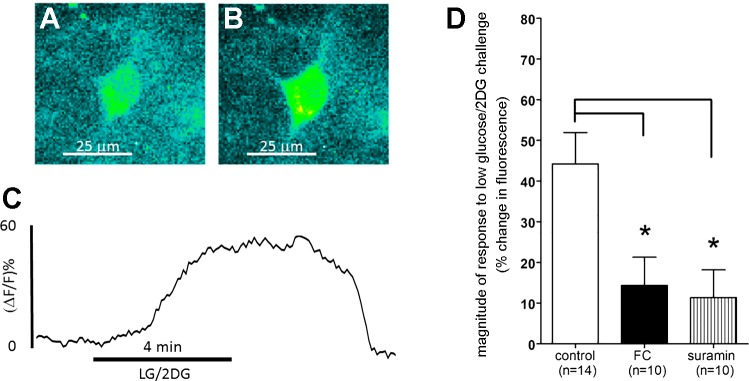
Tyrosine hydroxylase (TH)-GCaMP5 ventrolateral medulla (VLM) neuron response to glucoprivation stimulus. *A*: screenshot of VLM neuron before low-glucose/2-deoxyglucose (LG/2DG) challenge. *B*: screenshot of same neuron in *A* during LG/2DG stimulation. *C*: plot of change in fluorescence of VLM TH-GCaMP5 neuron over time in response to LG/2DG exposure. *D*: ~92% (13/14) of the viable TH-GCaMP5 VLM neurons demonstrated increased intracellular calcium in response to the glucoprivic challenge. Similar to the TH-GCaMP5 nucleus of the solitary tract neurons, VLM neuron activation by glucoprivation was essentially blocked by pretreatment with fluorocitrate (FC) or suramin [ANOVA: *F*_2,31_ = 6.5, *P* = 0.0043; Dunnett’s multiple-comparison post hoc test: *q* (FC) = 2.9; *q* (suramin) = 3.2, *P < 0.05]. Δ*F/F*, change in fluorescence intensity relative to baseline.

Approximately 92% (13/14) of the viable TH-GCaMP5 VLM neurons demonstrated increased intracellular calcium in response to the glucoprivic challenge (Fig. 8). Similar to the TH-GCaMP5 NST neurons, VLM neuron activation by glucoprivation was essentially blocked by pretreatment with FC or suramin [ANOVA: *F*_2,31_ = 6.5, *P* = 0.0043; Dunnett’s multiple-comparison post hoc test: *q* (FC) = 2.9, *q* (suramin) = 3.2, *P* < 0.05].

### Immunocytochemical Identification

**Fig. 9.**
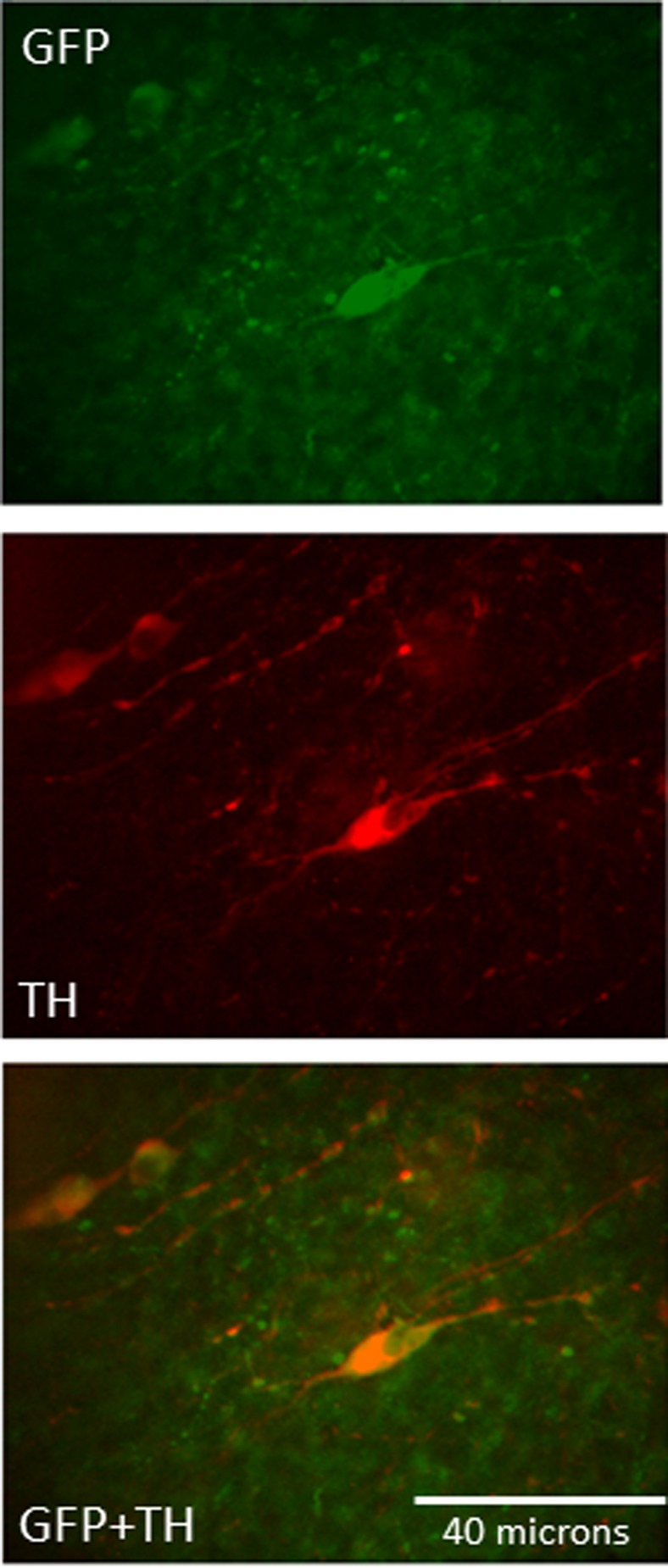
Immunohistochemical identification of tyrosine hydroxylase (TH)-GCaMP5 [via antibody for green fluorescent protein (GFP); green] and TH (via antibody for tyrosine hydroxylase; red) staining on nucleus of the solitary tract neurons at the rostrocaudal level of the area postrema. Colocalization of both antibodies appears as orange.

Fluorescently tagged monoclonal antibodies specific for TH or GFP (a component of GCaMP5) were used to verify that neurons expressing GCaMP5 were, indeed, CA neurons. NST neurons located within the histological sections containing the area postrema were evaluated using a Nikon E800 epifluorescence microscope equipped with a Hamamatsu ORCA charge-coupled device camera. Of the 434 NST neurons that were labeled by either antibody for GFP or antibody for TH, ~77% (333/434) were double labeled with both antibodies (Fig. 9); 12% (54/434) were only labeled by anti-GFP antibodies, and 11% (48/434) were tagged only for anti-TH antibodies.

## DISCUSSION

These studies demonstrate that a glucoprivic challenge induced by low glucose plus the antiglycolytic agent, 2DG, activates both astrocytes (~74%) and neurons (~67%) in the NST. On average, astrocyte responses precede those of neurons by >30 s. Pretreatment of brain slices with the astrocyte metabolic blocker, FC, abolished calcium responses to glucoprivation by both astrocytes and neurons but did not impair responses of either astrocytes or neurons to glutamate. In contrast, when slices were pretreated with the purinergic P2 receptor blocker, suramin, astrocytes were still activated by glucoprivic conditions, but neurons were not.

Experiments using TH-GCaMP5 mice revealed that ~90% of the NST and VLM CA neurons were robustly activated by glucoprivation; this neuronal activation appears to be astrocyte dependent. Finally, these brain slice imaging studies, in combination with pharmacological pretreatments, suggest that the connection between glucoprivation-responsive astrocytes and CA neurons is purinergic, favoring the P2Y_11_ receptor mechanism. The involvement of ATP as a potential signal from low glucose-sensitive astrocytes is reinforced by the finding that ATP robustly activates CA neurons in both the presence and absence of FC. These findings are consistent with previous in vivo studies in the rat (66), which showed that both localized and systemic reductions in glucose availability triggered increases in glycemia through an astrocyte-dependent, purinergic mechanism.

Several lines of evidence suggest astrocytic involvement in the detection of glucopenia. For example, systemic administration of the selective glial toxin methionine sulfoximine blocks 2DG-induced c-fos expression in NST neurons (79). Transgenic mice with a global knockout of GLUT2, a glucose transporter expressed by astrocytes and some neurons, are unable to increase glucagon secretion in response to a hypoglycemic challenge. Selective reexpression of GLUT2 in astrocytes, but not neurons, rescued this hypoglycemia defense mechanism (38), suggesting that astrocytes form an obligatory component of central nervous system control of glucagon secretion. In addition, responsiveness to glucoprivation has been demonstrated previously in astrocytes in the NST (40), where both focal and systemic glucoprivation activates astrocytes and increases blood glucose. The blood glucose response appears to require an obligatory astrocyte step involving purinergic gliotransmission (66). Taken together, these data suggest that astrocytes, activated by glucoprivation, communicate with hindbrain autonomic effector neurons to elicit at least some CRRs.

To test the hypothesis that glucoprivic activation of astrocytes secondarily excites NST neurons, we first used live cell calcium imaging to determine whether neurons in the NST are either intrinsically sensitive to glucoprivation or activated by astrocytes sensitive to glucoprivic conditions. Both neurons and astrocytes were prelabeled with the calcium reporter dye Cal-520, whereas astrocytes were identified by their selective uptake of SR101. Hence, we were able to monitor both NST astrocyte and neuronal responses to glucoprivation simultaneously. As seen in our previous studies (40), both astrocytes and neurons exhibited robust increases in intracellular calcium in response to glucoprivation. Moreover, the astrocyte calcium signaling, on average, preceded the neuronal calcium signal. Consistent with the hypothesis that glucoprivic activation of astrocytes is upstream of neuronal activation, we found that the astrocyte metabolic blocker, FC, eliminated the glucoprivation-induced increase in both astrocyte and neuronal calcium signals without blocking responses to other agonists such as glutamate or ATP. These results suggest that the glucoprivic activation of at least some NST neurons is dependent on astrocytes.

The specificity of action of FC in glial cells is supported by several, now classic, biochemical observations. First, glial cells avidly take up and metabolize FC and its metabolic precursor, fluoroacetate, whereas neurons do not (12, 46). FC is an aconitase inhibitor (12), and the metabolic result of aconitase inhibition in astrocytes is a rapid accumulation of citrate (8). Citrate is a well-known calcium chelator, and this is responsible for the rapid elimination of astrocyte calcium signaling by FC (12, 19, 30, 36, 54). This effect is not observed in neurons (77).

The effect of FC on astrocytes is analogous to the effect of tetrodotoxin (TTX) on neurons. FC selectively blocks a step in astrocyte metabolism; TTX blocks voltage-gated sodium channels. The end result is that each cell type ceases to signal activation. As a result, cell-cell communication is blocked. Both effects are reversible, and as a result, both drugs are very useful in vitro or ex vivo. Although such inactivation effects of FC and TTX are easily interpretable over the short term (i.e., minutes), long-term exposure, especially to FC, will lead to significant problems. A normal astrocyte function is to stabilize neuronal excitability and to provide metabolic support. Therefore, long-term exposure (i.e., hours) to FC can lead to astrocyte damage and a resultant hyperexcitability and cell death in neurons (20, 26, 30, 56). In vivo use of either drug must be approached with far more caution because of the practical problems of limiting effects to desired cell populations.

In our second series of experiments, we conducted live cell imaging on genetically identified CA neurons in the NST and VLM using TH-GCaMP5 transgenic mice. TH-GCaMP5-expressing neurons were vigorously activated by glucoprivic stimuli. Again, this activation appeared to be completely dependent on functional astrocytes as the astrocyte-specific inhibitor FC blocked the response (Figs. 6 and 8). The FC effect is not due to generalized damage to neurons as evidenced by their robust response to glutamate or ATP after FC treatment (Figs. 2 and 7; 66, 74).

There are several gliotransmitter pathways that can connect primary astrocyte chemosensor cells with the excitability of adjacent hindbrain autonomic control neurons. For example, glucoprivic conditions in the NST that ultimately trigger increased gastric motility appear to involve the release of adenosine onto inhibitory A_1_ receptors on distention-sensitive NST neurons (29, 40, 66). Astrocyte release of purines, particularly ATP and adenosine, are also implicated in the chemosensitivity of the ventral respiratory group to CO_2_ and acceleration of the respiratory rhythm (21, 35).

Glutamate has also been implicated as an autonomic gliotransmitter in models of trauma-induced gastric stasis (28). Thrombin, acting on astrocytes through proteinase-activated receptor 1, evokes a potent release of glutamate causing an amplification of the synaptic inputs from vagus to NST neurons through an NMDA mechanism (74). This causes NST hyperactivation resulting in inhibition of the adjacent dorsal motor nucleus gastric vagal efferent neurons (63).

The effects of selective glutamate and purine receptor antagonists used in this study suggest that the activation of CA NST neurons by glucoprivation-mediated gliotransmission involves activation of intracellular calcium release through P2 purinergic receptors, especially the P2Y_11_ receptor coupled with G_q_ protein-inositol (1,4,5)-trisphosphate mediated release of calcium stores (17). Our results also suggest the involvement of the adenosine A_2A_ receptor (which also triggers calcium release from the ER; 32). Also, purinergic gliotransmission as a mechanism to activate presympathetic CA neurons in the CRR mechanism is supported by the observation that these neurons are strongly activated by ATP under both control and FC pretreatment conditions (Fig. 7).

Similar to the TH-GCaMP5 cells in the NST, VLM CA neurons also were robustly activated by LG/2DG stimulation. However, the numbers of cells recorded were limited by the fact that relatively few such cells were visible because of the presence of heavily myelinated reticular fiber bundles coursing through the VLM. Unlike the effectively unmyelinated NST, where TH-GCaMP5-expressing cells were visible throughout the depth of the brain slice, only CA VLM neurons on the upper surface of the slice were available for study. In any event, this glucoprivation-driven response was also eliminated by pretreatment with FC and the P2 purine antagonist suramin. Thus, hindbrain CA neurons previously associated with the integrated control of CRRs appear to be activated by hindbrain glucoprivation-sensitive astrocytes utilizing purinergic (ATP) gliotransmission.

The details by which glucoprivic activation of astrocytes might be coupled to gliotransmission are unknown. Although there is evidence connecting increases in astrocytic intracellular calcium with gliotransmission utilizing mechanisms similar to those involved in neurotransmission (24), a connection between astrocyte glucose utilization, calcium signaling, and gliotransmission has yet to be made. However, astrocyte detection of glucoprivation appears to require GLUT2 (38). Reduced extracellular glucose availability translated to the intracellular milieu via GLUT2 yields a rapid decline in glycolysis. Astrocytes generate ATP primarily through glycolysis (31). Rapidly falling ATP could starve critical ion transporters of fuel, especially the calcium-ATPase pump in the ER, resulting in potential leakage of calcium from the ER into the cytoplasm. Alternatively, it has been shown that some cells may utilize specialized versions of GLUT2 possessing transduction capabilities. These “transceptors” might connect differential glucose flux through GLUT2 with mechanisms impacting calcium entry and release (22). Increases in astrocytic calcium could couple to gliotransmitter mechanisms releasing either glutamate or purines, signaling elements described above, that could alter astrocyte transmission. A reduction in glycolytic ATP could cause an overflow of adenosine independent of any calcium gliotransmission scheme (42, 72). At this point, any or all of the above mechanisms must remain theoretically equivalent candidates for coupling mechanisms.

### Perspectives and Significance

The mechanisms by which glucoprivic activation might be coupled to gliotransmission are unknown. Nevertheless, this report is consistent with the hypothesis that some CA neurons, potentially including those capable of initiating important CRRs, are activated by glucose deficit sensed and transmitted by astrocytes. It is widely recognized that hindbrain CA neurons have numerous functions, many of which are not glucoregulatory [for review, see Guyenet et al. (23)], and that some neurons that appear to be activated by glucose deficit are not TH positive. However, the results reported here extend the evidence that astrocytes are important regulators of hindbrain neuronal responses to metabolic challenge and suggest a potential role of astrocytes in eliciting protective responses to glucose deficit.

## GRANTS

This study was supported in part by National Institute of Diabetes and Digestive and Kidney Diseases (NIDDK) Grant DK-108765, American Diabetes Association Grant 1-15-JF-37, National Institute of General Medical Science Grant 1-U54-GM-104940, and NIDDK Grants DK-040498 and T32-DK-064584.

## DISCLOSURES

No conflicts of interest, financial or otherwise, are declared by the authors.

## AUTHOR CONTRIBUTIONS

R.C.R., D.H.M., S.R., and G.E.H. conceived and designed research; R.C.R., S.R., E.Q.-C., and G.E.H. performed experiments; R.C.R., S.R., and G.E.H. analyzed data; R.C.R., S.R., and G.E.H. interpreted results of experiments; R.C.R., S.R., and G.E.H. prepared figures; R.C.R., S.R., and G.E.H. drafted manuscript; R.C.R., S.R., and G.E.H. edited and revised manuscript; R.C.R., D.H.M., S.R., E.Q.-C., and G.E.H. approved final version of manuscript.
